# Understanding Post-Esophagectomy Complications and Their Management: The Early Complications

**DOI:** 10.3390/jcm12247622

**Published:** 2023-12-11

**Authors:** Jenifer Edmondson, John Hunter, Gennadiy Bakis, Amber O’Connor, Stephanie Wood, Alia P. Qureshi

**Affiliations:** Division of General Surgery, Oregon Health & Science University, Machall 3186, Portland, OR 97239, USA; edmondsj@ohsu.edu (J.E.);

**Keywords:** esophagectomy, post-operative complications, anastomotic leaks, anastomotic strictures, dysphagia, dumping syndrome, delayed gastric emptying

## Abstract

Esophagectomy is a technically complex operation performed for both benign and malignant esophageal disease. Medical and surgical advancements have led to improved outcomes in esophagectomy patients over the past several decades; however, surgeons must remain vigilant as complications happen often and can be severe. Post-esophagectomy complications can be grouped into early and late categories. The aim of this review is to discuss the early complications of esophagectomy along with their risk factors, work-up, and management strategies with special attention given to anastomotic leaks.

## 1. Introduction

Significant improvement in mortality and morbidity associated with esophagectomy has occurred over the past few decades because of improved efficiency in pre-operative care, surgical techniques, and post-operative care. Esophagectomy may be the treatment of choice for esophageal pathology that includes both benign and malignant pathology, stricture, caustic injury, achalasia, Barrett’s esophagus, and esophageal cancer. Esophageal malignancy is currently the most common indication for esophagectomy [1]. A study investigating the ESODATA database performed by Low and colleagues (2019) indicated that the majority of esophagectomies were performed for malignant disease (95.6%) [1]. Accordingly, esophageal adenocarcinoma is the most common esophageal malignancy followed by squamous cell carcinoma (most common in Eastern Europe and Asia), according to the Surveillance, Epidemiology and End Results (SEER) Program [2,3]. Per Dubecz and colleagues [4], as many as 55–70% of patients with esophageal cancer in the SEER Database have potentially resectable disease, but only 44% of these patients undergo esophagectomy. This may in part be due to clinicians’ and patients’ perceptions that esophageal cancer surgery is more palliative than it is curative, and esophagectomy is wrought with complications. Despite improvement in esophageal cancer treatment, surgery, and associated morbidity and mortality over the past several decades, this stigma remains [4].

The Esophagectomy Complications Consensus Group (ECCG) was formed in 2011 [5] to establish a standardization of data collection for post-esophagectomy complications. The ECCG established a basic list of complications that should be included in databases as well as key standard definitions for anastomotic leak, conduit necrosis, chyle leak, and recurrent nerve palsy [5]. Twenty-four ECCG centers across 14 countries prospectively collected data into an online database (ESODATA) from January 2015 to December 2016, and the overall complication incidence was found to be 59% [1]. The most common esophagectomy complications within the ECCG database included pneumonia, atrial dysrhythmias, and anastomotic leaks with incidence rates of 14.6%, 14.5%, and 11.4%, respectively. The less common complications included chyle leak and recurrent nerve injury with respective incidence rates of 4.7% and 4.2%. Each of these complications can contribute to the morbidity and mortality of esophagectomy, as well as affect post-esophagectomy quality of life. Here, we review some of the early complications associated with esophagectomy and the treatment strategies to manage them.

## 2. Methods

The aim of this review was to create a comprehensive piece on post-esophagectomy complications including their risk factors, work-up, and management strategies with special attention given to anastomotic leaks. Studies involving the ESODATA database helped demonstrate the various complications that may happen following esophagectomy, and these were the complications that were selected for this review. A systematic review was performed utilizing PRISMA 2020 criteria. PubMed was utilized to search for studies related to post-esophagectomy complications and their management. The terms used for the search included: esophagectomy, post-esophagectomy complications, anastomotic leaks, post-op management, atrial fibrillation, dysphagia, post-esophagectomy pulmonary complications, esophagectomy nerve injury, delayed gastric emptying, and post-esophagectomy reflux were utilized.

## 3. Esophagectomy: Early and Late Complications

Esophagectomy-related complications can occur both early and late (Table 1), with the overall rate of post-esophagectomy complications estimated to be about 59% [1]. Typically, early complications include anastomotic leak, atrial dysrhythmias, pneumonia/aspiration, chylothorax, and recurrent nerve palsy. Late complications include dysphagia/stricture, delayed gastric emptying, bile reflux, dumping syndrome, and malabsorption. Anastomotic leaks, however, can present both as early complications, suggesting technical etiology, as well as late complications, suggesting a delayed healing or malperfusion type of etiology. In this paper, we set out to review early esophagectomy-related complications and in so doing, include their incidence, risk factors, clinical presentation, diagnostic work-up, and treatment strategies as well as their respective impact on long-term outcomes.

### 3.1. Anastomotic Leaks

#### 3.1.1. Incidence and Definitions

Without a doubt, anastomotic leak is the most feared complication of esophagectomies. Per the ESODATA database, the incidence rate of anastomotic leak is 11.4% [1]. For, clarity, the different classification systems for esophageal leaks are reviewed here. The Oesophago-Gastric Anastomosis Audit (OGAA) sought to find the rates of anastomotic complications and calculated a leak rate of 14.2% [6]. Both studies used the ECCG definitions of anastomotic leak when analyzing their data sets. The ECCG definition of anastomotic leak is a “full thickness gastro-intestinal defect involving the esophagus, anastomosis, staple line, or conduit, irrespective of presentation or method of identification” [5]. The ECCG further divides anastomotic leaks and conduit necrosis into three types based on the type of intervention required to manage the leak or necrosis (Table 2). In the OGAA study, 15.7% of patients with anastomotic leak also had conduit necrosis associated with the leak, and 83% of patients with conduit necrosis had an associated anastomotic leak. The rates of conduit necrosis increased significantly with increasing anastomotic leak type [6]. 

Another classification system for anastomotic leak was developed by Bruce and colleagues in 2001 [7] using the definition from the Surgical Infection Study Group: a leak of luminal contents from a surgical joint between two hollow viscera [8]. The Surgical Infection Study Group is a UK multidisciplinary group and, in addition to defining anastomotic leak, also recognizes a category of subclinical leak that is defined by the escape of luminal contents from the anastomosis, which is detected with imaging in the absence of clinical symptoms. Using these definitions, Bruce and colleagues classified grades of anastomotic leak into categories including radiological, clinical minor, and clinical major [7] (Table 3). This classification schema uses a combination of clinical and radiologic indications to categorize anastomotic leaks in contrast with the ECCG classification schema, which uses the level of intervention needed to treat anastomotic leaks for their categorization. Both classification systems help attribute a sense of the severity of individual anastomotic leaks. Broadly speaking, the two classification systems complement each other in that the Bruce and Colleagues grading system attributes a clinical severity indicator that correlates with the ECCG classification of anastomotic leak management (ECCG Correlate Column Table 3).

The OGAA prospective cohort study described an overall anastomotic leak rate of 14.2% and further investigated leak rates for intra-thoracic versus cervical anastomoses. The leak rate for intra-thoracic anastomoses was 12.2%, and the leak rate for cervical anastomoses was 20.1% [6]. Though intra-thoracic anastomotic leaks occurred at a lower rate than cervical leaks, the intra-thoracic leaks were of significantly higher morbidity (ECCG Type II and III), had a higher likelihood of needing surgical intervention, and were associated with a longer length of hospital stay and ICU stay compared with cervical anastomotic leaks, which occurred at a higher incidence but were less morbid.

#### 3.1.2. Risk Factors and Prevention

Risk factors can be categorized into tissue factors, patient factors, and technical factors. Within each category, there may be risk factors that are modifiable and risk factors that are not. Risk factors that are intrinsic to the esophagus (tissue factors) and not modifiable include the absence of serosa and the longitudinal muscle fibers of the esophagus. The lack of serosa and the presence of longitudinal muscle fibers means the remnant esophagus is delicate. This especially rings true when one considers suture holding in the esophagus compared with other tissues with serosa and circular muscle fibers like the stomach and small bowel. These tissue factors can mean a more tenuous anastomosis, especially when combined with other risk factors.

There are several patient factors that are modifiable, and thus, the prevention of anastomotic leaks should focus on these modifiable factors. Patient risk factors can be further categorized into preoperative factors and perioperative factors. Some modifiable preoperative factors to consider are malnutrition, diabetes, cardiovascular conditions, respiratory insufficiency, and smoking and drinking habits, as well as applied neoadjuvant therapies. These factors can contribute to poor or prolonged healing and are also risk factors for other post-operative complications. Efforts at preoperative optimization should consider these factors and may reduce the risk of anastomotic leaks and other complications. Two notable perioperative factors that may contribute to anastomotic leak risk are hypotension and hypoxemia. Hypotension contributes to poor tissue perfusion, thus increasing the risk of conduit tissue ischemia and necrosis. The use of vasopressors in the perioperative period may also negatively impact tissue perfusion and oxygenation but may be necessary for blood pressure support. Hypoxemia negatively impacts tissue oxygenation and thus can also contribute to conduit ischemia and necrosis [9]. 

The reconstruction aspect of esophagectomy is a technical factor to consider and is not modifiable. Not only is there extensive dissection, but the mobilization of the neo-esophagus, most often the stomach [10], requires the sacrifice of part of the gastric blood supply. Consequently, there is potential for ischemia and necrosis that can then result in anastomotic breakdown and leakage when meticulous attention is not paid to detailed dissection. Another technical factor that may contribute to the risk of anastomotic leak is the type of esophagectomy. Different types of esophagectomy are associated with different incidences of anastomotic leak. Ozawa and colleagues [11] performed a review of 48 studies with more than 50 patients who underwent minimally invasive esophagectomy to further evaluate the incidence of post-esophagectomy complications. In their review, the overall incidence of anastomotic leak was 9.3% for minimally invasive esophagectomy (MIE). They further evaluated the incidence of leak by type of MIE and found values of 7.8% for McKeown MIE, 10% for Ivor Lewis MIE, 18.5% for robotic-assisted McKeown MIE, 6% for robotic-assisted Ivor Lewis MIE, and 9.8% for trans-mediastinal esophagectomy. It is also important to note that the severity of presentation of anastomotic leak differs between McKeown esophagectomy and Ivor Lewis esophagectomy due to the location of the anastomosis (cervical anastomosis in McKeown versus thoracic anastomosis in Ivor Lewis). A thoracic anastomotic leak is more likely to have a more severe and dramatic presentation due to a leak of contents into the mediastinum/thoracic cavity, whereas a leak from a cervical anastomosis is less likely to be as severe (drainage from cervical incision, neck abscess, etc.) [11].

Other technical factors have been studied to assess their effectiveness in preventing anastomotic leaks, which include the manner of anastomosis (hand-sewn versus stapled, circular versus triangular, end-to-end versus end-to-side) and gastric ischemic preconditioning (the practice of ligating short gastric and left gastric vessels to redistribute gastric blood supply with the aim to improve perfusion to the future anastomotic site) [12,13]. However, no significant decrease or difference in leak rate has been identified. Prevention measures should focus on preoperative optimization and conditioning, intraoperative care that minimizes hypotension, hypoxemia, and blood loss, and maneuvers that cause direct trauma to the conduit [10]. Post-operative preventive maneuvers should focus on resuming early enteral nutrition, emphasizing good pulmonary toilet, and preventing hypotension and hypoxemia [14].

#### 3.1.3. Presentation

Depending on the severity of anastomotic leak, the presentation of this problem may vary from having minimal clinical signs to having fulminant sepsis. A patient may have a subclinical leak where they display no clinical signs of tachycardia, fever, leukocytosis, or changes in drain output. These subclinical leaks are found on routine post-operative contrast esophagograms or CT imaging that is obtained for other reasons. Some patients may present with subtle clinical signs such as tachycardia, atrial fibrillation, and elevated inflammatory markers on blood tests [10]. In some cases, atrial fibrillation may be the only sign of anastomotic leak, so any post-esophagectomy patient with atrial fibrillation within the first week and after post-operative fluid shifts occurred should be at high suspicion for leak. Post-operative fluid shifts occur within the first 3 post-operative days; therefore, atrial fibrillation may be expected in the first 3 days after esophagectomy. However, any onset of atrial fibrillation after post-operative day 3 should increase the clinician’s index of suspicion for a possible anastomotic leak or other post-operative complications. For larger leaks, the signs may be more drastic and can create a septic picture. Additionally, there may be increased contents from surgical drain or neck incision if present. If the anastomosis is in the thoracic cavity, leaks may present with oxygenation issues, hydropneumothorax, effusions, and the need for pulmonary support [15]. Some key pieces of information to remember are that cervical anastomoses have a higher incidence than thoracic anastomoses [1], the presentation of a leak can occur on a spectrum from asymptomatic to needing an ICU level of care with ventilatory and ionotropic support, leaks can be large (high output) but may not have a drastic septic picture if adequately drained via surgical drain or neck incision or if well-contained, and the clinician’s level of suspicion for leak needs to remain high with even the most subtle of clinical signs.

#### 3.1.4. Diagnostic Work-Up

There is no gold standard for diagnosing a post-esophagectomy anastomotic leak. There are, however, various diagnostic tools and considerations that can aid in the diagnosis, and these include clinical signs, drain fluid, laboratory tests, endoscopic examination, contrast esophagograms, and CT imaging. The clinical signs discussed previously can certainly support the diagnosis of anastomotic leak. A patient with tachycardia, fever, leukocytosis, and elevated inflammatory markers (such as C-reactive protein) may have anastomotic leak, but these same signs could be present with other post-operative complications as well. Saliva or gastric contents in the surgical drain or draining from the neck incision are obvious indicators of a leak. Drain fluid can be tested for amylase levels and high amylase would support leak diagnosis. It has been proposed that routine drain amylase testing on post-operative day 4 could serve as an early leak detection [16]. Such early detection can aid in expediting intervention and possibly preventing further associated complications. 

Upper endoscopy is a diagnostic modality that can be considered when diagnosing leaks. In addition to finding the leak, endoscopy can also assess the integrity of the conduit, which can help determine which patients may need surgical revision. Understandably, many may be hesitant to pursue upper endoscopy due to the fear of disrupting the anastomosis. Endoscopy in this setting should be performed by an experienced endoscopist, and only gentle and progressive insufflation should be used. In their porcine studies, Raman and colleagues [17] were able to demonstrate that even at maximal insufflation, the anastomotic endoluminal pressure did not exceed 8.7 cm H_2_O pressure, which is far below what is needed to disrupt anastomosis or disturb tissue perfusion. The theoretical risk of further disruption of the anastomosis is a valid concern but should be weighed against the benefits of endoscopy in a patient with a suspected anastomotic leak. Endoscopy can serve to confirm the presence of a leak, further characterize the size and severity, and assess the viability of the conduit. All these factors should be considered to determine if the patient would be best served with surgical, endoscopic, or medical interventions. 

Contrast esophagogram is a common tool modality for the diagnostic work-up of anastomotic leak and entails using a water-soluble contrast agent followed by thin barium (improves sensitivity for leak detection). It is important to assess the patient’s swallowing function prior to performing this test as the contrast agent can cause chemical pneumonitis if aspirated. Using a water-soluble contrast (e.g., gastrograffin) first is a safe maneuver because if there is a leak appreciated with gastrograffin, the diagnosis of the leak is confirmed. If no leak is detected, thin barium is then utilized because it improves the sensitivity for leak detection. Water-soluble contrast is used first because it is thought to be safer if there is a leak with contrast entering the mediastinum and/or thoracic cavity. Barium is utilized following a negative water-soluble contrast study because it is more radiopaque and better detects smaller leaks. Unfortunately, even with a negative contrast study, a leak cannot be ruled out. The sensitivity for leak detection is about 93% for intra-thoracic anastomoses and even lower (33–52%) for cervical anastomoses [10]. However, it is highly specific, so a positive study does confirm the diagnosis of a leak. If a contrast esophagogram is negative for a leak but there remains a high level of suspicion for a leak, further work-up should be sought. 

CT imaging is a quick and non-invasive modality that can help diagnose an anastomotic leak, and it can provide additional information on the surrounding tissues and structures of the neck, thorax, and abdomen. A CT scan may be able to detect a leak and appreciate peri-anastomotic fluid collections, pulmonary effusions, mediastinal fluid, etc. However, even with oral contrast use, a leak may not be detected. Additionally, findings of peri-anastomotic free air and fluid may be consistent with expected post-operative changes. However, findings of mediastinal fluid and air, anastomotic wall discontinuity, and fistula are significantly associated with anastomotic leak [10]. Another benefit of CT imaging is that it may identify fluid collections that can be targeted for drainage.

#### 3.1.5. Treatment Strategies

General management strategies are discussed in this section. A more in-depth discussion of endoscopic versus interventional versus surgical treatment strategies is provided in a later section of this review. Treatment options vary from little change in medical therapy to surgical intervention. Treatment modalities include observation, medication (octreotide, proton-pump inhibitors, antibiotics), drainage (IR drainage, wound drainage, nasogastric drainage), endoscopic stenting, endoscopic vacuum-assisted closure, and surgical repair/diversion/re-anastomosis. When considering treatment strategies for anastomotic leaks, factors that should be considered include leak location, leak size, conduit drainage, clinical stability of the patient, and availability of interventional resources [18]. 

Small occult leaks found on routine post-operative esophagograms can be treated by delaying oral food intake. Small symptomatic leaks in stable patients can be treated by restricting oral intake while supporting nutrition with enteral nutrition if access is in place; otherwise, parenteral nutrition can be used. Any infectious signs should prompt the initiation of antibiotic therapy. Any sizable fluid collections should be drained for infectious control. Other medical therapies that can be initiated include the initiation of somatostatin to decrease gastric acid secretion and proton pump inhibitors (decreases gastric acid production) to promote anastomotic healing [14]. For cervical anastomoses, if drainage is present around the incision, the incision should be opened to allow for adequate drainage and then allowed to heal by secondary intention. 

Larger leaks, especially those not contained will likely require more invasive treatment. Interventional radiology may be needed to drain thoracic fluid collections. Endoscopy should be considered to evaluate the viability of the conduit mucosa. If necrotic and ischemic tissue is present, surgical intervention to revise or divert the anastomosis may be necessary. Some leaks may be amenable to endoscopic stenting of the anastomosis or endoscopic vacuum-assisted closure of the defect, which will be discussed in a later section. However, if the patient is in or heading toward fulminant sepsis, the patient needs to return to the operating room to evaluate the anastomosis and debride any necrotic tissue. Diverting esophagostomy may also be necessary, and if a feeding jejunostomy tube is not already present, it should be considered upon return to the operating room.

The clinician must be aware that the complication of anastomotic leak may precipitate other complications including empyema and fistula to the airways (both tracheal and bronchial). Prompt, adequate drainage is necessary to help prevent these complications, but is also a key aspect of treatment if these complications of anastomotic leaks occur. Esophagobronchial fistulas following esophagectomy have a reported incidence of 0.25–3% and mortality rates of up to 67%, according to a literature review performed by Sato and colleagues [19]. 

### 3.2. Atrial Fibrillation

#### 3.2.1. Incidence and Etiology

Atrial fibrillation and other tachyarrhythmias are common post-operative complications. Across ECCG centers, the incidence of atrial arrhythmias occurred at 14.5%, making atrial fibrillation and pneumonia (14.5%) the most common post-esophagectomy complications [1]. Atrial fibrillation in the first three post-operative days is most likely secondary to expected post-surgical fluid shifts. After post-operative day 3, atrial fibrillation should be considered as a clinical sign of another complication, especially anastomotic leaks. Atrial fibrillation can be the first and only sign of an underlying septic etiology: anastomotic leak, pneumonia, wound infection, urosepsis, etc. Therefore, the clinician’s index of suspicion should be high that there could be another problem when atrial fibrillation is encountered in this setting. 

#### 3.2.2. Risk Factors

Risk factors for post-operative atrial fibrillation can be divided into pre-operative, intra-operative, and post-operative groups (Table 4). Pre-operative risk factors include old age (70+), cardiac factors, diabetes, and obesity [20]. Some of these risk factors are modifiable, which can serve as a prevention strategy in the pre-operative care setting. Some peri-operative risk factors can be mitigated with the judicious use of fluids, minimizing blood loss, encouraging early ambulation, and good pulmonary toileting.

#### 3.2.3. Presentation and Diagnostic Work-Up

Atrial fibrillation can present as new onset tachycardia, sudden onset of dyspnea, lightheadedness or dizziness, diaphoresis, chest pain, and other changes in vital signs such as desaturation and hypotension. Frequently, though, patients may be asymptomatic, and atrial fibrillation is found after work-up of irregular tachycardia. If atrial fibrillation is suspected in a post-operative patient, the initial steps that should be taken include new vital signs and bedside examination of the patient, and an electrocardiogram should be obtained immediately to confirm the diagnosis and an assessment of whether the patient is symptomatic from the arrhythmia (dyspnea, chest pain, hypotension, diaphoretic, etc.). Laboratory tests should be ordered and should include an assessment of electrolyte levels, cardiac enzymes, complete blood count (look for leukocytosis or anemia), and plus/minus inflammatory markers. Chest X-ray should also be considered to evaluate possible effusion or pneumonia that could have precipitated the arrhythmia.

#### 3.2.4. Treatment

Management of post-operative atrial fibrillation is guided by the underlying etiology. The main goal in treating atrial fibrillation is to maintain hemodynamic stability. Treatment approaches include rhythm control versus rate control and consideration of cardioversion therapy if needed. Rate control (a heart rate of 90–115) can be achieved with a beta-blocker (metoprolol, esmolol, or propranolol), non-dihydropyridine calcium channel blocker (verapamil or diltiazem), or digoxin. Beta-blockers tend to be the preferred agents and most effective at controlling the ventricular response in the setting of atrial fibrillation but may be contraindicated in severe asthmatics (beta-blockers may cause bronchospasm) and atrioventricular conduction disorders (high degree AV block is an absolute contraindication) as beta-blockers are AV nodal blocking agents and can worsen such conduction disorders and may even progress to complete heart block. Calcium channel blockers should be avoided in AV conduction disorders as well for this same reason [20]. Digoxin may be helpful in the setting of congestive heart failure, but cardiology consultation would be recommended in this setting. 

If rate control is not achieved, the patient may have to be transferred to the intensive care unit or an acute care floor to receive amiodarone. Amiodarone is an antiarrhythmic drug that works primarily by blocking potassium channels but also has a blocking effect on sodium and calcium channels, as well as at the beta- and alpha-adrenergic receptors. Our institution is currently investigating the prophylactic use of amiodarone for perioperative atrial fibrillation in minimally invasive esophagectomy patients.

Most cases of post-operative atrial fibrillation spontaneously convert to normal sinus rhythm within 24 h. Until then, supportive measures include repletion of electrolytes, encouraging ambulation, and good pulmonary toilet. Underlying causes should be investigated and treated appropriately. If a patient becomes hemodynamically compromised or is not responsive to pharmacologic therapy, electrical cardioversion may be necessary. If atrial fibrillation is persistent (>48 h), anticoagulation therapy should be considered if clinically safe to do so, as there is an increased risk of cerebrovascular incidents with persistent atrial fibrillation [20]. 

### 3.3. Pulmonary Complications

#### 3.3.1. Incidence and Risk Factors

Pulmonary complications occur more often than any other post-esophagectomy complication. According to the ECCG standardization of complications reporting, pulmonary complications include pneumonia, pleural effusion, pneumothorax, atelectasis, mucous plugging, respiratory failure, acute respiratory distress syndrome, acute aspiration, tracheobronchial injury, and prolonged air-leak [5]. The reported incidence of post-operative pulmonary complications is between 8% and 36% [10]. Pneumonia is the most common pulmonary complication with an incidence of 14.6% [1] according to Low and colleagues’ analysis of data from the ESODATA database. 

Risk factors for post-operative pulmonary complications can be categorized into pre-operative factors, technical factors, and post-operative factors. Preoperative risk factors are age (especially >70 years of age), nutritional status, underlying pulmonary disease (COPD, tuberculosis, lung fibrosis, etc.), and poor baseline pulmonary function. In their series of 421 patients, Law and colleagues [21] demonstrated that increased age over 70 conferred a twice as high risk for developing post-operative pneumonia with an associated mortality risk that was elevated fourfold. Smoking habits and alcohol use also contribute to risk. Smoking cessation one month prior to an operation may reduce rates of pulmonary complications [10]. Pre-operative pulmonary optimization can help mitigate some of these risks. Pulmonary rehabilitation can include breathing and cough exercises, spirometry, and the use of expectorants and bronchodilators.

Technical risk factors include the location of a tumor, surgical approach, blood loss, and duration of the surgery. Tumors located above the tracheal bifurcation were associated with the risk of pneumonia in Law’s 421-patient series [21]. A multicenter trial [18,22] found that pulmonary complications were less likely when thoracotomy was not used and when patients were able to be extubated immediately following surgery. This same trial also found that pneumonia was less common with MIE versus open esophagectomy and more common when a pyloric drainage procedure was not performed. These intraoperative risk factors indicate that minimally invasive techniques and maneuvers that minimize blood loss and surgical duration may aid in the prevention of pneumonia. 

Post-operative risk factors that contribute to pulmonary complications include vocal cord paralysis, recurrent laryngeal nerve palsy, swallowing problems, poor pulmonary hygiene, poor pain control, and post-operative respiratory muscle dysfunction. Swallowing problems and dysphagia are common causes of respiratory complications in the post-esophagectomy setting. Swallowing and vocal cord dysfunction are commonly thought to be caused by injury to the recurrent laryngeal nerve (RLN) during surgery. This can consequently lead to aspiration and other pulmonary complications including pneumonia. It was reported that 50% of patients with vocal cord paralysis suffered from respiratory complications [10]. If vocal cord paralysis or RLN injury is suspected, prompt work-up and therapy is warranted to prevent further complication and improve outcomes (dysphagia, voice hoarseness, and aspiration). Poor pulmonary toileting and pain control can contribute to the development of atelectasis, mucous plugging, and pneumonia. Encouraging ambulation, spirometry use, and deep breathing exercises can help mitigate this risk. Good pain control is a key factor in patients being able to perform these tasks. Adequate post-operative analgesia should be achieved with a multimodal approach to include consideration of epidural analgesia. 

#### 3.3.2. Presentation and Diagnostic Work-Up

Pneumonia and other post-operative pulmonary complications can present in several ways and with different severity. Pneumonia may be first suspected with a persistent and possibly productive cough that may be accompanied by fever and leukocytosis. There may be changes to vital signs that include tachycardia (possibly atrial fibrillation), increased respiratory rate, and lower oxygen saturation. The patient may complain of dyspnea and chest pain. Physical exam may reveal rales, rhonchi, or wheezing. Other pulmonary complications may be present that can contribute to the development of pneumonia such as atelectasis, aspiration, and mucous plugging.

If pneumonia or other pulmonary problems are suspected, a work-up should include a focused physical exam of the chest and swallowing function. A chest radiograph should be obtained to make the diagnosis clear. Other diagnostic adjuncts that are helpful include blood tests (looking for leukocytosis, elevated inflammatory markers, and blood cultures if febrile) and CT imaging if the pneumonia is suspected to be complicated or secondary to another cause such as anastomotic leak. If the etiology of the pneumonia is suspected to be secondary to vocal cord paralysis, delayed gastric emptying, or leak, a further work-up should be pursued to evaluate these potential etiologies.

#### 3.3.3. Treatment

Management of post-operative pulmonary complications may require a multidisciplinary approach. As stated previously, it is important to work up underlying etiologies and treat them appropriately. Antibiotics are needed in cases of pneumonia, empyema, or leaks. Some patients may require mechanical ventilatory support in the intensive care unit. Pre-operative factors such as the patient’s comorbidities are also likely to be at play and should be considered when considering management strategies, e.g., patients with COPD may require steroids if they experience a post-operative exacerbation. Consultation with respiratory therapists for aggressive pulmonary toilet and speech–language pathologists (especially in cases of vocal cord dysfunction or dysphagia) can also be helpful depending on the clinical picture. Supportive measures that should be encouraged if feasible for the patient are early ambulation, early extubation, multimodal pain control, incentive spirometry use, and aggressive pulmonary hygiene.

## 4. Strategies for Management of Anastomotic Leaks

Anastomotic leak is the most dreaded complication of esophagectomy. In the past two decades, there have been advances in treatment modalities for managing anastomotic leaks, but prior to this time, operative re-exploration was the preferred management strategy. Unfortunately, re-exploration is associated with significant in-hospital mortality (50%) [23,24]. There is no gold standard on how to manage anastomotic leaks following esophagectomy; however, there are multiple treatment modalities that may be used to construct a management strategy that is tailored to each individual patient. 

The treatment strategies for managing minor anastomotic leaks and severe or catastrophic leaks are clear. Minor leaks generally do not require intensive or invasive treatments. Catastrophic leaks with hemodynamic instability and a severely compromised anastomosis or conduit require surgery. The management strategy for anastomotic leaks that fall between these two extremes is less clear. Broadly speaking, management goals include sepsis and source control, sufficient drainage of leaks, and preventing further mediastinal contamination. Treatment options to accomplish these goals vary widely from conservative therapies with enteral support, chest tubes, and drainage tubes to aggressive surgical interventions with thoracotomy, primary repair/reconstruction of the anastomosis, conduit take down, and cervical esophagostomy. The available treatment modalities can be grouped into conservative measures and interventional therapies (drains), endoscopic interventions, and surgical treatments. Table 5 summarizes and compares these treatment modalities. Next, we discuss some of the available options within each of these groups.

### 4.1. Non-Surgical and Interventional Therapies

Non-surgical measures for anastomotic leaks focus on nutritional support, infection treatment, and wound care. It is preferential that nutrition comes from an enteral source via a nasogastric tube or jejunostomy tube. Even in patients with asymptomatic leaks, most surgeons opt for nil per os until the leak is resolved. Until the patient can have oral nutrition, their nutrition must be supported with feeds by a feeding tube or parenteral nutrition if necessary. If a feeding tube is not already in place, options include the endoscopic placement of a nasogastric tube or the surgical placement of a jejunostomy feeding tube. In some cases, the patient may have hemodynamic instability and may be in too fragile of a state to tolerate these procedures. In these cases, the patient should be supported with parenteral nutrition until they are more stable and enteral access can be established.

Infection control and wound care measures include intravenous antibiotics, opening the cervical wound (if applicable), and performing wound care to the area. In some cases of cervical anastomotic leaks, there may be drainage into the mediastinum, causing a systemic inflammatory response that may be severe. In such cases, mediastinal drainage should be considered. Interventional drainage of the mediastinum, perianastomotic abscesses, empyema, etc., should be considered if present. These measures serve to control sepsis, sufficiently drain the area of the leak, and help prevent further contamination, all of which are imperative for the successful treatment of anastomotic leaks. Depending on the size and severity of the leak, non-surgical measures may be the only treatment needed to heal the anastomotic leak. In more severe cases, these measures are still important aspects of care to promote healing and sepsis control. 

### 4.2. Endoscopic Interventions

Several endoscopic interventions are available that may be used to manage leaks. Endoscopic interventions for the treatment of post-esophagectomy anastomotic leaks are relatively new, and no standard guideline exists that outlines how to implement these types of interventions. Some of the endoscopic treatments available include endoscopic vacuum therapy, endoscopic stents, endoscopic clips, and fibrin glue. These interventions can be used separately, in combination with one another, or in combination with surgical therapies depending on the individual factors at play. However, it must be noted that ischemia of the gastric conduit precludes the use of endoscopic therapies.

#### 4.2.1. Endoscopic Vacuum Therapy

Vacuum-assisted closure therapy is a well-known modality for the treatment of extracorporeal traumatic and surgical wounds. Wiedenhagen and colleagues first describe intracorporeal use for anastomotic leaks following rectal surgery in 2008 [25]. The basic principles of vacuum-assisted closure therapy include decreasing bacterial burden, necrotic tissue, and local edema, as well as promoting local perfusion and tissue granulation with the use of negative pressure therapy over a polyurethane sponge. These principles hold true no matter the location of use. Specific to the upper gastrointestinal tract, the literature provides several case reports and series, as well as a few reviews on the use of endoscopic vacuum therapy for esophageal anastomotic leaks and perforation [23,24,26,27,28,29,30,31,32,33]. 

##### Technical Details of Endoscopic Vacuum Therapy

There is no commercial system available in the United States specific to EVT for esophageal anastomotic leakages. Some of the available reviews on EVT described the use of the Endo-Sponge system from Germany, which was initially for rectal use. A polyurethane sponge is modified to fit into the cavity (if present) or to adequately cover the anastomotic defect. The procedure is performed under general anesthesia. For critically ill patients who are on mechanical ventilation in the intensive care unit, this procedure could be performed at the bedside with sedation. 

The procedure can vary depending on whether there is a wound cavity present (intracavitary vacuum therapy versus intraluminal vacuum therapy) [29,31,32]. The procedure starts with inserting the endoscope into the esophagus and carefully evaluating the conduit and anastomosis. If a cavity is present, it may be intubated with the scope to help determine the size of the cavity and therefore the size to which the sponge must be trimmed [26]. The sponge is then fixed to the distal end of a nasogastric tube that has already been placed through the nasal cavity with a suture. The sponge is then placed with endoscopic guidance into the esophagus and defect. Ahrens described the use of an over tube that is placed over the endoscope and into the wound cavity where the sponge connected to the nasogastric tube is inserted through the over tube and into the cavity with a pusher device. The over tube is then removed, and the sponge positioning is checked with a repeat endoscopy [26]. Others describe placing the sponge under direct endoscopic visualization with the aid of endoscopic forceps [23,24,28,32]. Once the sponge is in a good position, the nasogastric tube is placed to negative pressure (suction). The cavity (if intracavitary) will collapse around the sponge and tubing once negative pressure therapy is started. The negative pressure setting varies in the literature. Some describe a setting of continuous negative pressure of 70–80 mmHg [24,26], and others describe a setting of negative 100–125 mmHg [23,28,29,30,31,32]. EVT can work in this location because of the intrapleural negative pressure that helps add to the vacuum of the system without adding any additional sealing [23]. EVT creates a pressure gradient allowing for intraluminal drainage of secretions. 

If there is no enteral access, endoscopic placement of a nasogastric/nasoduodenal tube can be safely performed during this procedure without further injury or compromise of the anastomosis. The feeding tube can be placed via the contralateral nostril from the EVT suction tube. Most of the literature reviewed supported changing the sponge every three days [23,28,32,34]. Some of the literature also noted that the time between sponge changes could expand over time to every 5 days or twice per week depending on the healing progression of the anastomotic defect [26,27,29]. 

At each sponge change, the anastomotic defect is endoscopically assessed for the progression of healing. The new sponge would have to be tailored to shape and size with each subsequent change (ideally, needing to be trimmed smaller with each change). Sometimes intracavitary EVT would be switched to intraluminal EVT with closure of the cavity after granulating in. There is no standardization for when to stop EVT. Schorsch terminated EVT once stable granulation formed a contamination barrier, and the wound was “self-cleaning” by the follow-up endoscopy [32]. Schniewind and colleagues stopped EVT when the diameter of the wound cavity was 2 cm or less [24]. Ahrens stopped EVT once the wound cavity was smaller than a 1 cm radius by 2 cm depth [26]. Other authors described stopping therapy once the defect size was too small for further sponge changes and epithelium was lining the defect. For some, closure may be completed with the use of an over-the-scope clip to seal the small, residual defect [29]. Once the defect is fully healed and EVT has stopped, there is usually a follow-up endoscopy to ensure complete healing. Injection of contrast into the defect area during EGD or performing an esophagogram after EVT removal can assess the success of EVT.

##### Advantages and Limitations

There are several benefits to using endoscopic vacuum therapy in the treatment of esophageal anastomotic leakage. One of the more important benefits is that it addresses one of the key tenets for managing anastomotic leaks, controlling leaks, and preventing further contamination. EVT provides local septic control. The pressure gradient that it creates causes preferential drainage of infectious material and secretions into the conduit lumen. EVT allows for the evacuation of necrotic debris and interstitial edema that could impede anastomotic healing. EVT promotes blood flow and granulation formation, which is critical to healing the anastomotic defect [33]. Another key advantage is that endoscopy allows for the direct visualization of the anastomosis and conduit to assess vascular perfusion and the size of the defect. EVT allows for regular visualization of the anastomosis with each sponge change to assess the progress of healing and shrinkage of the anastomotic defect. Ahrens and colleagues [26] noted a decrease in the white blood cell count and CRP levels as well as an improvement in APACHE II scores in patients who were started on EVT for esophageal anastomotic leaks. This indicates the effectiveness of local septic control with EVT because it controls leakage and evacuates necrotic and infectious debris.

Combination therapy with percutaneous drains and surgical interventions is also an option. There are cases of leaks that are not contained, and a mediastinal washout may be warranted. Empyema may be present and require drainage or evacuation. Sometimes there is a need for anastomotic surgical revision, especially if obvious ischemia and necrosis are present. EVT may still be used in these cases to help promote wound cleanliness, granulation, and healing. EVT can be used even with revision surgery of an anastomosis to promote blood flow to the area and promote anastomotic healing [23].

There are a few advantages that EVT has over other endoscopic therapies for anastomotic leaks. Other endoscopic therapies such as stents, glues, and clips may seal the leak but do not drain or debride the paraoesophageal tissue surrounding the defect. These other endoscopic options do not serve to control local septic conditions but rather serve to limit further mediastinal contamination. EVT, on the other hand, serves to control local sepsis and prevent further contamination. If a wound cavity is present, these endoscopic measures do not serve a purpose in healing and collapsing the cavity. When it comes to defects in the more proximal esophagus, stent placement is extremely difficult, but EVT is still a treatment option in this area. There are a few studies that compare EVT to stent therapy for the treatment of esophageal anastomotic leaks. One retrospective study, performed by Schniewind and colleagues [24], investigated 62 patients with anastomotic leaks following esophageal resection. There were three treatment groups: EVT, stent, surgery, and conservative management. Systemically ill patients matched for APACHE II scores were compared, and the EVT group had a lower mortality rate (12.3%) compared with the stent (83%) and surgery (50%) groups. Another study from Korea conducted by Hwang and colleagues [35] was a retrospective analysis of outcomes in 18 patients with anastomotic leaks following esophageal surgery. All patients (7) in the EVT group were treated successfully, but only 63.6% (7 of 11) of patients in the stent group had successful treatment. They also noted a lower complication rate (0%) in the EVT group compared with the stent group (54.5%).

EVT does have its limitations. In the past, EVT was not performed if ischemia was present at the anastomosis or gastric conduit, nor was it used if mediastinal or systemic sepsis was present. As EVT has become more common, some of these limitations have been overcome [23,33]. Currently, EVT should not be used if complete anastomotic dehiscence is present, if the gastric conduit is necrosed, or if the defect or cavity is near a major vascular structure, as catastrophic damage to a major vessel caused by EVT is a concern [33]. For EVT to work, there must be local perfusion. EVT can promote perfusion in the wound bed, but adequate perfusion must be present when initiating therapy. EVT may be successful in some cases where there is some ischemia or necrosis present but is not an appropriate therapy if it is extensive, involves the entire anastomosis (dehiscence), or the conduit is compromised [29]. 

Another consideration is that EVT requires multiple procedures and is performed under sedation or general anesthesia. In critically ill patients on mechanical ventilation, EVT procedures would not add additional stress since they are already on sedation. However, there is a resource burden incurred as sponge changes happen every 3 to 5 days and are performed by an advanced endoscopist. Depending on the status of the patient, an anesthesiologist may be needed for sedation or general anesthesia. Frequent sponge changes may serve as an advantage though because they enable regular viewing of the wound bed. There is also the argument that repeat endoscopic procedures are less traumatic for the patient than a reoperation. One additional consideration is that there is no commercially available device in the US, and the vacuum sponges are used “off label”. 

##### Outcomes and Complications

Some minor complications related to EVT therapy include sponge dislocations, minor mucosal bleeding from granulation ingrowth into the sponge, and nasogastric tube-related issues (discomfort, clogging, dislodgement). Sponge migration can happen, especially with intraluminal placement, and can be avoided with bridling of the NGT. Some minor erosion of the mucosa with EVT may be seen, which affects mucosa in direct contact with the sponge. This erosion is temporary with regeneration of normal mucosa seen within a few days after the removal of the sponge [31]. A feared, but rather rare, complication is major hemorrhage because of enteroaortic fistula, fistulization to the great vessels and heart chambers, or pseudoaneurysm of nearby major vascular structures related to ongoing inflammatory processes. Such major bleeding events can be fatal and have been reported in the literature [29,33]. Other complications associated with EVT are dysphagia and stricture. In their review, Kuehn and colleagues [27] noted six case series that evaluated the incidence of stricture in post-EVT follow-up. Combined, they found a total of 12 patients out of 157 that were affected by strictures (7.6%). All strictures were resolved with endoscopic dilation. It is important to note that this association is likely related to an anastomotic leak complication rather than a direct complication of EVT. 

#### 4.2.2. Stents

Previously, endoscopic stenting was used primarily for non-surgical patients for palliation from obstructing esophageal cancer or malignant fistulas. More recently stents have been used in the treatment of anastomotic leaks with the goals of covering the defect, earlier resumption of oral intake, and avoiding surgical intervention [36]. Several retrospective studies and reviews have been published on the efficacy of stent therapy for anastomotic leaks. Stents may be used in cervical or intrathoracic anastomoses, though they seem to be more efficacious for intrathoracic anastomotic leaks [37,38]. In one review, thirty-seven patients had stents placed for either anastomotic leak or perforation [39]. This review noted that esophageal stents can play an important role in managing leaks; however, the success of stent therapy is dependent on appropriate source control (drainage of abscesses). Success rates of stent therapy (full anastomotic healing/closure) vary from 59% to 100% [37,38,39,40,41] in the reviewed literature. 

##### General Technical Steps and Details

Stent placement is performed under general anesthesia or conscious sedation under endoscopic visualization. If stricture is present, the use of fluoroscopy may be warranted. The procedure starts with the endoscopic evaluation of the anastomosis to assess the extent of the defect and mark relevant anatomic landmarks. A stent is selected based on the appropriate diameter and length. Landing zones (proximal and distal) are planned out. A guidewire is then placed under direct visualization. The stent is then placed over the wire and deployed under fluoroscopic guidance. Some endoscopists may perform balloon dilation to fully deploy the stent. Post-procedure radiographs are taken to confirm the good placement and deployment of the stent [36].

There are three types of endoscopic stents that can be used: biodegradable stents, self-expandable non-metal stents, and self-expandable metal stents. Self-expandable metal stents are the most common and have a lower migration rate compared with plastic stents [36,42]. Some endoscopists may use endoscopic suturing or clipping of the stent to prevent migration. Biodegradable stents maintain their radial force for about 6 weeks and then degrade over a period of 6–24 weeks. Acid suppression is required with biodegradable stent use, as an acidic environment would accelerate degradation [36]. A follow-up esophagogram in 24–48 h has been described to assess stent sealing of the defect and a follow-up endoscopic stent removal and contrast swallow study is performed after 4–6 weeks [39].

##### Advantages and Disadvantages of Stent Therapy

Stent placement is a familiar and readily available treatment modality. Like other endoscopic therapies, stent placement allows for the endoscopic evaluation of the anastomosis and mucosa to assess the viability of the conduit at the time of placement and subsequent removal. During stent placement, endoscopic drainage procedures may be performed to aid in sepsis control. A feeding tube can also be placed through the placed stent after deployment under direct endoscopic guidance. A key advantage of stent therapy is that it can help avoid reoperation. In their series, Feith et al. [41] noted an in-hospital mortality rate of 9% in the stent treatment cohort. However, one must consider that a contraindication to stent therapy would be extensive ischemia of the conduit or necrotic anastomosis. These cases necessitate operative therapy and tend to lead to sicker patients, so it is not surprising that reoperation would be associated with higher mortality and morbidity rates. 

Stent therapy does have disadvantages. There is a risk of improper stent placement, which only adds to the risk of complications without the benefit of source control. Risks of improper stent placement include erosion, perpetuation of the leak, aspiration, and obstruction. Stents may not be tolerated by some patients, especially in those with high anastomosis. Stent therapy also requires at least one follow-up endoscopic procedure for removal, but there is also the risk that the leak will not be fully healed at that point and another stent may have to be placed [39]. Stent therapy cannot be used in cases of extensive conduit ischemia or necrosis. Stent therapy also does not address abscesses or empyema that require drainage and antimicrobial therapy. Sometimes, a peri-anastomotic abscess can be endoscopically drained, but this is not feasible for other locations. Leaks along the gastric conduit staple line are not amenable to stent therapy given the large diameter of the conduit, which would not allow for adequate sealing of the leak [37].

##### Factors to Weigh When Considering Stent Therapy

Factors that can impact the success of stent therapy include leakage size, time to leak diagnosis and treatment, and degree of contamination/mediastinal infection. The larger the leak, the more difficult it will be to control with stent therapy. Furthermore, the longer it takes to diagnose anastomotic leak and initiate therapy, the more difficult it will be for a stent to seal the leak because of the inflamed and friable tissue. Additionally, stent therapy should only be performed in patients with adequate perfusion of the anastomosis [41]. If the conduit is compromised, it will not be able to heal even if a stent is able to seal the leak. 

Other key factors that need to be weighed include the luminal diameter and vertical orientation of the conduit. If the diameter of the esophagus or conduit is too large, a stent will not be able to seal off the leak. If the conduit is angled, the distal aspect of a stent may be occluded and could potentially lead to perforation of the conduit. Leaks within 2 cm of the cricopharyngeus muscle are not amenable to stent therapy because stenting across this muscle or just underneath it can be very uncomfortable for the patient.

##### Complications of Stent Therapy

The most common complication of stent therapy is stent dislocation, with an occurrence rate between 16.2% and 53% in the literature [39,41]. Stent migration can present early or late and usually only requires endoscopic intervention for retrieval and replacement. Stent dislocation may require surgical intervention in the event of obstruction or intestinal perforation. Feith et al. noted that 3% of patients who underwent stent therapy for anastomotic leak or esophageal perforation needed surgical intervention (all needed exploratory laparotomy) due to stent dislocation. The type of conduit may be a risk factor for stent dislocation as well. Both Feith and Langer [41,43] reported stent dislocations in 100% of esophagocolonostomies compared with esophagogastrostomies (49% and 44% respectively) and esohpagojejunostomies (61% and 20% respectively). Other minor complications include pain/discomfort, nausea, and reflux (both bile and acid). 

More serious complications include stent erosion/perforation, leak enlargement, and gastro-aortic fistula. These occur rarely (1–3%) [38,39,41]. Stent erosion and leak enlargement likely require surgical intervention and will prolong the hospital stay. Stent erosion into the aorta is a lethal complication and is described by Schweigert and colleagues in their institutional review [38]. One of twelve patients treated with stent therapy for esophageal anastomotic leaks died of sudden massive hemorrhage because of stent erosion and fistulization into the thoracic aorta. This occurred 17 days after stent insertion. Schweigert noted that septic vascular erosion is a known, although rare, complication of intrathoracic anastomotic leaks and does not require the presence of a stent to happen. Nevertheless, stents are still considered a risk factor for vascular erosion.

Anastomotic strictures are also seen after stent therapy. Feith noted that 12% of patients treated with stents for anastomotic leaks developed symptomatic anastomotic strictures, and an additional 26% were found to have asymptomatic anastomotic strictures on follow-up endoscopy [41]. All anastomotic strictures in this series were easily treated with endoscopic intervention (mainly bougie dilations). Anastomotic strictures may be a complication of anastomotic leak rather than stent therapy, but this can be difficult to discern.

#### 4.2.3. Other Endoscopic Options: Endoclips and Fibrin Glue and Vicryl Plugs

##### Endoclips

Endoscopic clipping can be used for several different problems including anastomotic leaks, perforation, and fistulas. Endoclips were first used for closing anastomotic leaks in 1995 [36]. There are two types of clips that can be used: through-the-scope clips (TTSCs) and over-the-scope clips (OTSCs). Generally, a TTSC is used for smaller defect closure less than 1–2 cm in diameter, whereas an OTSC is used for larger diameter defects (up to 3 cm in diameter) [44]. TTSCs dislodge spontaneously in about 2–4 weeks but can also prematurely dislodge and cause a recurrence of leaks. It appears that OTSCs are used more often than TTSCs for the management of anastomotic leaks [45]. An OTSC is much more difficult to remove, so if not placed properly the first time or if it fails to close the defect, removing an OTSC can be problematic and requires the use of an argon plasma device, bipolar cutting device to fracture the clip, or cold saline to increase malleability [36]. Kobara and colleagues performed a review of over 1500 cases over a 9-year period involving OTSC use and concluded that the best setting for OTSC use is within 1 week of diagnosis, low level of fibrosis, and size between 10 and 30 mm [46]. Endoclips are limited in closing large defects (>2 cm). One case report [44] describes using a hybrid technique with OTSC and a detachable snare, where multiple clips are placed around the periphery of the defect followed by applying the detachable snare around the endoclips and tightening in a purse-string manner to close the defect. This technique is likely more successful in an optimal environment with healthy tissue at the defect edges and minimal contamination and fibrosis. 

##### Fibrin Glue and Vicryl Plugs

The use of glues to seal anastomotic leaks and fistulas has variable success rates in the literature ranging from 56% to 97% [36]. The data in the literature for fibrin glue sealant is heterogeneous, and there is no clear set of indications for the use of fibrin glue for the treatment of anastomotic leaks. Fibrin glue can be considered for the use of small defects in a clean environment free of necrotic debris. A case series out of Germany [47] investigated the use of fibrin glue or the combination of fibrin glue and Vicryl mesh plugs for treating upper gastrointestinal fistulas and leaks in 39 patients. Fifteen of these cases were treated with a combination of a Vicryl plug and fibrin glue. Briefly, the procedure for this treatment entails endoscopic evaluation of the defect and lavage of the cavity with 0.9% NaCl until the cavity appeared clean and had an entry site of 1.5 cm. The leak site is then filled with an absorbable Vicryl mesh, preferably with large pores (>2 mm) to allow for absorption of the fibrin glue that is applied afterward (2–3 mL). Out of the fifteen patients who underwent combined Vicryl plug and fibrin glue therapy, 13 healed completely (87%). This technique requires clean surfaces free of necrotic and infectious debris. This technique is not recommended for patients with fulminant anastomotic leaks (sepsis, necrotic anastomosis, ischemic conduit, etc.). This technique may be used in combination with other endoscopic therapies. A stent may be placed over the site, which would allow for early oral nutritional intake. Also, with the use of endoscopic vacuum therapy, the defect may decrease to such a small size that is not amenable to further sponge therapy, but the size and “clean” nature of the defect may be a perfect setting for fibrin glue application. 

### 4.3. Surgical Options

There are several surgical options available for the management of esophageal anastomotic leaks; however, there is no set standard for when surgical intervention should be performed and what type of surgical intervention. Such a standard would be difficult to discern as the management of leaks should be individualized for each patient per their specific symptoms and severity of the leak. Generally, surgical intervention is indicated for symptomatic leaks that are not contained (especially those that are intrathoracic), leaks that fail to improve with more conservative therapies, and worsening sepsis in the setting of anastomotic leak. Patients that have non-contained leaks that are septic must be adequately and expeditiously resuscitated and should be immediately re-explored. During reoperation, the pleural cavity and mediastinum should be debrided of non-viable tissue and collections drained, and any necrotic stomach should be resected [48,49].

Further intraoperative management is dependent on the viability of the esophagus, conduit, and hemodynamic stability of the patient. If there is extensive ischemia or necrosis of the gastric conduit, especially in the setting of hemodynamic instability, resection of the conduit with the creation of cervical esophagostomy is the likely preferred approach. There may be focal necrosis or ischemia along the anastomosis that is amenable to local resection followed by re-anastomosis. The margins of the anastomosis should be clean and healthy if attempting this solution. Some surgeons may also consider reinforcing the repair with a flap (pleura, omentum, pericardial fat, pericardium, pedicled chest wall muscle) [50]. They may also consider evaluating the gastric staple line, as leaks can occur in this area as well. The success of reoperation is dependent on controlling the leak during the reoperation. 

Page and colleagues [49] describe an algorithm for how they manage surgical treatment of anastomotic leak. Within this algorithm, the type of surgical management is dependent on the length and viability of the remaining gastric tube. If the gastric conduit was healthy (focal identifiable leak), the affected tissue was resected, and if the patient’s condition was favorable, immediate re-anastomosis was performed. In cases where there was extensive gastric necrosis, a cervical end-esophagostomy was performed and was later followed by delayed colonic reconstruction. In cases with some gastric necrosis but a sufficient length of the remaining viable conduit, the creation of a “double barreled” cervical esophagogastric stoma was considered. In this surgical approach, the remainder of the viable stomach is drawn into the neck between the trachea and carotid sheath and is sutured to adjacent strap muscles or skin along with the distal esophagus. Adjacent borders of the stomach and esophagus can be sutured together, and this serves as the posterior portion of the future anastomosis. This method provides the benefit of being able to evaluate the gastric mucosa directly at the bedside or even with endoscopy via the gastric stoma to ensure healthy tissue prior to completing the anastomosis.

Given the history of the high mortality rate associated with reoperation for anastomotic leaks, there has been a push in the past two decades to find other management strategies. However, one must consider that the high mortality associated with reoperation is likely reflective of the more complicated and severe conditions of patients who are indicated for operative intervention. These patients tend to be in more critical conditions with sepsis and multi-organ failure compared with patients who undergo less invasive therapies. Therefore, the high mortality in reoperation should be thought of as consequential to the severity of the anastomotic complication rather than with the reoperation itself. Though other less invasive treatment modalities are of significant value, it is very important to scrutinize the clinical situation and determine as early as possible if surgery would be the best intervention.

## 5. Conclusions

Esophagectomy is a complex surgical procedure performed for either benign or malignant esophageal pathology. Regardless of the indication for esophagectomy, the procedure is technically advanced and may be fraught with complications, necessitating thoughtful surgical planning and precise technical execution. It is important for surgeons to be aware of the possible complications and their management.

## Figures and Tables

**Table 1:** Common Esophagectomy Complications.

Early Complications	Late Complications	Complications That May Present Early or Late
Anastomotic Leak Atrial Dysrhythmias Pneumonia/Aspiration Chylothorax Recurrent Nerve Palsy	Dysphagia Stricture Delayed Gastric Emptying Bile Reflux Dumping syndrome Malabsorption	Dysphagia Delayed Gastric Emptying Reflux

**Table 2:** ECCG Classification Types of Anastomotic Leaks.

ECCG Classification Types	Anastomotic Leaks	Conduit Necrosis
Type I	No change in therapy needed or treated medically with diet modification	Focal necrosis found on endoscopy
Type II	Interventional therapy such as drain, stent placement, or bedside drainage of wound	Focal necrosis on endoscopy that is not associated with leak, but requires interventional/surgical therapy that does not involve esophageal diversion
Type III	Requires surgical therapy	Extensive necrosis that requires conduit resection and diversion

**Table 3:** Anastomotic Leak Grades: Radiological, Clinical Minor, and Clinical Major by Bruce and Colleagues (2001) and ECCG Correlate [5,7].

Grade	Signs/Symptoms	Management	ECCG Classification Correlate
Radiological	Detected only on routine imaging. No clinical signs	No change.	Type I—No change in therapy or treated with diet modification
Clinical Minor	Drain or wound contain luminal contents causing local inflammation ○ May have fever (>38 °C) ○ Leukocytosis Leak may be detected on imaging studies	No change, but may have prolonged hospital stay and/or delay in resuming oral intake.	Type I–II—Above and/or Interventional therapy needed (drain, stent, bedside wound drainage)
Clinical Major	As above for clinical minor Leak may be detected on imaging studies Severe disruption to anastomosis.	Change in management and intervention required.	Type II–III—Above and/or surgical therapy needed

**Table 4:** Risk Factors for post-op atrial fibrillation in the esophagectomy patient.

Preoperative Risk Factors	Intraoperative Risk Factors	Postoperative Risk Factors
Age > 70 years old Enlarged left atrium Left ventricular hypertrophy Hypertension Diabetes Obesity	Thoracic Surgery Acute Changes in blood volume Operative Time Cardiac Injury	Volume Overload Electrolyte disturbances Pulmonary complications Sepsis

**Table 5:** Summary of Treatment Modalities for Anastomotic Leaks.

Treatment Modality	Indications and Goals of Treatment	Effort	Treatment Success	Limitations
Conservative Therapies (Non—interventional)	Should be initiated for any severity of leak. Non-interventional approach can be attempted for minor clinical leaks or small radiographic leaks in hemodynamically stable patients. Goal is to minimize leak and contamination and allow for anastomotic healing.	Easy: NPO, TPN, use of feeding tube if present, antibiotics	Critical aspect of care for all grades of anastomotic leaks. Can be successful as only treatment modality for clinically minor leaks.	Need parenteral or distal enteral access if not already present. Otherwise, no major limitations.
Interventional Therapies (Drains and Chest Tubes)	Indicated in those with undrained abscesses and/or collections, pleural effusions, empyema, and/or mediastinal contamination. Goals of Therapy: sepsis control, drainage, and prevention of further mediastinal contamination.	Some effort required with bedside procedures (for chest tube placement and/or opening surgical wound) and/or use of interventional radiology for drain placement.	Necessary aspect of management for some clinically minor and all clinically major leaks.	No major limitations. Surgeons should be able to perform these bedside procedures. Limitation with drain placement could be IR availability and/or need to place drains surgically.
Endoscopic: EVT	Can be considered in those with healthy conduit and defect >2 cm. Can be placed into cavities associated with anastomotic leak site. Can assist with drainage and sepsis control (local source control). Promotes ingrowth of granulation tissue to shrink the defect.	Requires an advanced endoscopist who is experienced with EVT (not readily available in many medical centers in the United States).	Associated with lower complication rates and mortality rates in a few small studies when compared to stents and surgery.	Experienced advanced endoscopists are not widely available in all areas. Requires serial procedures for sponge exchanges. Not indicated for management ischemic/necrotic conduit.
Endoscopic: Stent	Can be considered for smaller leaks and especially in those without an associated cavity. Goal is to seal of the area and allow healing without further leak contamination.	Requires availability of Therapeutic Endoscopy	Success rates vary from 59–100%. Success rates largely dependent on appropriate source control.	Stent migration is the most common complication. Requires at least 2 procedures (placement and removal). Should not be used if there is focal ischemia/necrosis of the anastomosis.
Endoscopic: Clips	Can be useful in very small defects or those defects that have shrunk to a small size following EVT. Goal is to seal the defect to prevent further contamination.	Requires availability of Therapeutic Endoscopy	No great data in literature for overall success rate of this therapy in management of anastomotic leaks, but literature indicates higher success of this modality when treating small defects (< 1–2 cm).	Should not be used for defects >2 cm. The surrounding tissue must be healthy (no focal ischemia/necrosis).
Surgery	Should be performed in those patients with leaks accompanied by severe sepsis and/or hemodynamic instability that is not responsive to resuscitative efforts. Indicated in those patients who fail less invasive therapies.	ChallengingAny surgeon performing esophagectomy should be prepared to perform reoperation for diversion, re-anastomosis, drainage, and/or establishing new conduit.	Associated with higher morbidity and mortality rates. This is likely related to the illness severity of patients who undergo surgery (severe sepsis, hemodynamic instability, multiorgan failure, etc.)	Operative staff/support availability. Patients indicated for surgical intervention are likely critically ill and will require immense post-operative support and rehabilitation.
